# The Most Effective Pollinators Predict Intraspecific Floral Color Diversity Across an Edaphic Matrix

**DOI:** 10.1002/ece3.74360

**Published:** 2026-09-17

**Authors:** Katherine E. Riddle, Taylor N. Sherer, Jenna McCarty, Stacy Taylor‐Bennetts, Matthew H. Koski

**Affiliations:** ^1^ Department of Biological Sciences Clemson University Clemson South Carolina USA; ^2^ Department of Environmental Horticulture University of Florida Gainesville Florida USA; ^3^ School for Environment and Sustainability, University of Michigan Ann Arbor Michigan USA; ^4^ Department of Biological Sciences Augusta University Augusta Georgia USA

**Keywords:** floral evolution, flower color, Grant–Stebbins model, *Phacelia dubia*, pollination effectiveness, pollinator‐mediated selection

## Abstract

Colonization of new environments can spur floral evolution when plants encounter novel selective regimes such as unique pollinator communities. Although edaphic transitions are frequently associated with floral diversification, the extent to which pollinators mediate such divergence remains poorly understood. A generalist‐pollinated granite outcrop endemic, *Phacelia dubia* , colonized ruderal meadow‐like roadsides and lawns within the last ~60 years in the Southeastern United States. We quantified flower color (blue chroma) and size in outcrops and meadows in the field and greenhouse, characterized pollinator communities, and measured pollination efficiencies of common pollinators. We tested whether edaphic transitions restructure generalist pollination systems and contribute to floral divergence consistent with the Grant–Stebbins model of floral evolution. Flowers in natal outcrops were darker on average, and more variable among populations than meadows—outcrop populations were either predominantly purple or white‐flowered, while meadows were consistently white‐flowered. There was a strong genetic contribution to color divergence among populations. Family‐level pollinator composition differed between habitats and correlated with color, especially when weighted by pollinator efficiencies. Pairwise pollinator community distance was positively correlated with flower color distance, and this was strongest for between‐habitat comparisons. Spatial variation in the relative abundance of the most effective pollinators (Megachilidae, Apidae) predicted color variation better than more common but less effective pollinators (Andrenidae, Syrphidae). Our study supports that edaphic transitions can expose plant populations to novel pollinator communities and create opportunities for floral evolution. Geographic variation in the most effective pollinators predicted flower color variation, providing support for the Grant–Stebbins “most effective pollinator” principle in a plant with generalized insect pollination.

## Graphical abstract

A generalist‐pollinated plant endemic to rock outcrops recently expanded to weedy roadside meadow populations where it exhibited reduced flower color variation and less pigmented flowers. A relationship between flower color and pollinator community composition, and an especially strong link between color and relative abundance of the most effective pollinators support that pollinators may contribute to floral color evolution. Together, the study suggests exposure to different pollinator communities following an edaphic transition may shape floral color evolution in a generalist‐pollinated plant.

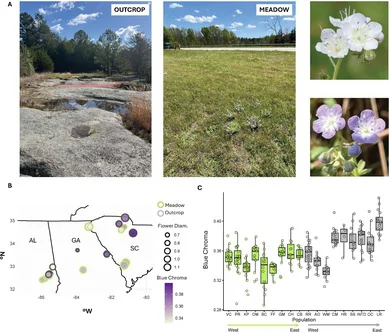

## 1 Introduction

Geographic heterogeneity in species interactions fuels phenotypic diversification via the process of coevolution (Thompson 2019). Studies of plant–pollinator interactions provide several examples of how spatial variation in mutualisms structures floral phenotypic diversity across species ranges (Anderson and Johnson 2008; Gómez et al. 2009; Wenzell et al. 2023; Moir et al. 2025) and contributes to the speciation process (Bradshaw and Schemske 2003; Kay and Sargent 2009; Van der Niet et al. 2014). In most cases, local floral ecotypes possess a suite of traits that match the morphology and sensory preference of the most locally abundant and/or effective pollinator (Anderson et al. 2010; Cosacov et al. 2014; Peter and Johnson 2014). While pollinator‐mediated floral diversification is best studied in plants with specialized pollination, generalist pollination is a common strategy (Waser et al. 1996) and variance in relative pollinator abundance has been linked with floral variation in generalist‐pollinated plants (Gómez et al. 2014; Wenzell et al. 2023; Moir et al. 2025). In either case, these studies draw on principles of the Grant–Stebbins model of floral evolution (Johnson 2006), which argues that floral traits evolve in response to the most frequent and effective pollinators in a given population. Extensions of the Grant–Stebbins model stress that floral traits evolve to maximize fitness rather than match a single pollinator type (Aigner 2001; Kay and Anderson 2025; Newman et al. 2026). Therefore, floral traits can be adapted to several pollinators, such that pollination generalism is not always an unstable intermediate strategy on the evolutionary trajectory toward specialization.

Grant and Grant (1965) first proposed that the most frequent pollinator of a given plant species should be the primary agent of selection molding its floral phenotype. Stebbins (1970) later elaborated that a given pollinator's pollen transfer capacity should also be considered, and that flowers should adapt to the pollinator that is the most effective at pollen transfer and most frequent. Linking both relative pollinator abundance and pollination effectiveness with floral traits across species' ranges is uncommon (however see Wenzell et al. 2024; Moir et al. 2025) but has the potential to reveal nuanced patterns of covariation between pollination communities and floral phenotypes consistent with the Grant–Stebbins model (Kay and Anderson 2025). For example, geographic variation in the abundance of pollinators that contribute little to pollination may not correlate with floral variation because they are weak agents of selection. Conversely, geographic variation in the most effective pollinator(s) should correspond with floral variation if they exert stronger selection.

Floral color is frequently studied in the context of the Grant–Stebbins model because it can experience divergent pollinator‐mediated selection across space (Streisfeld and Kohn 2007; Hopkins et al. 2012) and contribute to speciation (e.g., Bradshaw and Schemske 2003). Classic examples of this process involve spatial variation in the frequency of broadly defined pollinator taxonomic groups like hummingbirds versus bees which possess disparate color visual systems and drive divergent selection for flower color. Color visual systems however can vary substantially within a given broad taxonomic group. For instance, for two Hymenopteran taxa of similar size— *Apis mellifera* (Apidae) and *Osmia rufa* (Megachilidae)—the peak spectral sensitivity of photoreceptors differ by 8, 15, and 13 nm for UV, blue, and green photoreceptors, respectively (van der Kooi et al. 2021). Therefore, it may be important to characterize pollinators to taxonomic levels lower than broad functional group (e.g., large bee, small bee; Fenster et al. 2004; Wenzell et al. 2023; Torres‐Vanegas et al. 2026) when considering how the pollinator community may covary with flower color. This may be especially true for highly generalized insect‐pollinated plants that do not experience very dramatic pollinator shifts across space.

Expansion into new habitats either through range shifts (Kelly and Goulden 2008) or human‐mediated introductions (Richardson and Pyšek 2012) may contribute to floral diversification by exposing plants to novel pollinator communities (Richman et al. 2020). Given that floral phenotypes can rapidly adapt to novel pollinator‐mediated selection imposed experimentally (Gervasi and Schiestl 2017), it follows that floral traits in populations that colonize habitats with novel pollinator communities could rapidly adapt in the wild. Indeed, intercontinental expansion of *Digitalis purpurea* from its native European range to the Americas in the 1800s exposed it to bird pollination, which altered selection on floral morphology and contributed to flower size differences between the native and naturalized ranges (Mackin et al. 2021). Expansion into novel edaphic habitats, characterized by distinct soil conditions, has the potential to drive floral diversification (Brady et al. 2005; Wright and Stanton 2007; Rajakaruna 2018; Grossenbacher et al. 2026), but the role of pollinators as the primary ecological driver of floral diversity between edaphic habitats is poorly understood. Edaphic environments can, however, differ strongly in pollination environments. For instance, visitation rates and pollinator functional groups differed between serpentine and non‐serpentine *Mimulus guttatus* (Meindl et al. 2013), and *Astragulus purpurinus* was primarily Bumblebee‐pollinated in alpine meadows but mason bee‐pollinated in sandy sites (Deng et al. 2025). Moreover, comparative evidence from the South African flora supports that combined edaphic and pollination system shifts are important for driving sympatric speciation (van der Niet et al. 2006). Thus, plant systems that have experienced edaphic shifts are well suited to reveal insights into the role of pollinator shifts in shaping floral phenotypic evolution.

In this study, we assess tenets of the Grant–Stebbins model of floral evolution in a system that experienced a recent edaphic shift. *Phacelia dubia* (Hydrophyllaceae) is an annual plant endemic to granite rock outcrops in the Southeastern United States that has become “weedy” in grassy meadow‐like roadsides and lawns where it appeared between 1972 and 1984 (Taylor‐Bennetts and Levy 2024). We sampled several outcrop and meadow populations to evaluate whether flower color and size differ between habitat types and conducted a common garden study to test the degree to which population‐level differences are genetically based. We collected pollinators visiting *P. dubia* across three flowering seasons and identified them to family to test whether more recently colonized meadows experience novel pollinator communities, and whether pollinator composition covaries with floral phenotypes. For the seven dominant pollinator families, we further quantified pollen carrying capacity and fidelity to *P. dubia* . We predicted (1) flower traits should exhibit genetic differences between habitat types if expansion to meadows was associated with floral evolution, (2) pollinator community composition should differ between habitat types, and be linked with floral trait variation if pollinators contribute to floral evolution, and (3) geographic variation in more effective pollinators should explain more floral variation than that of less effective pollinators.

## 2 Materials and Methods

### 2.1 Study System

**FIGURE 1:**
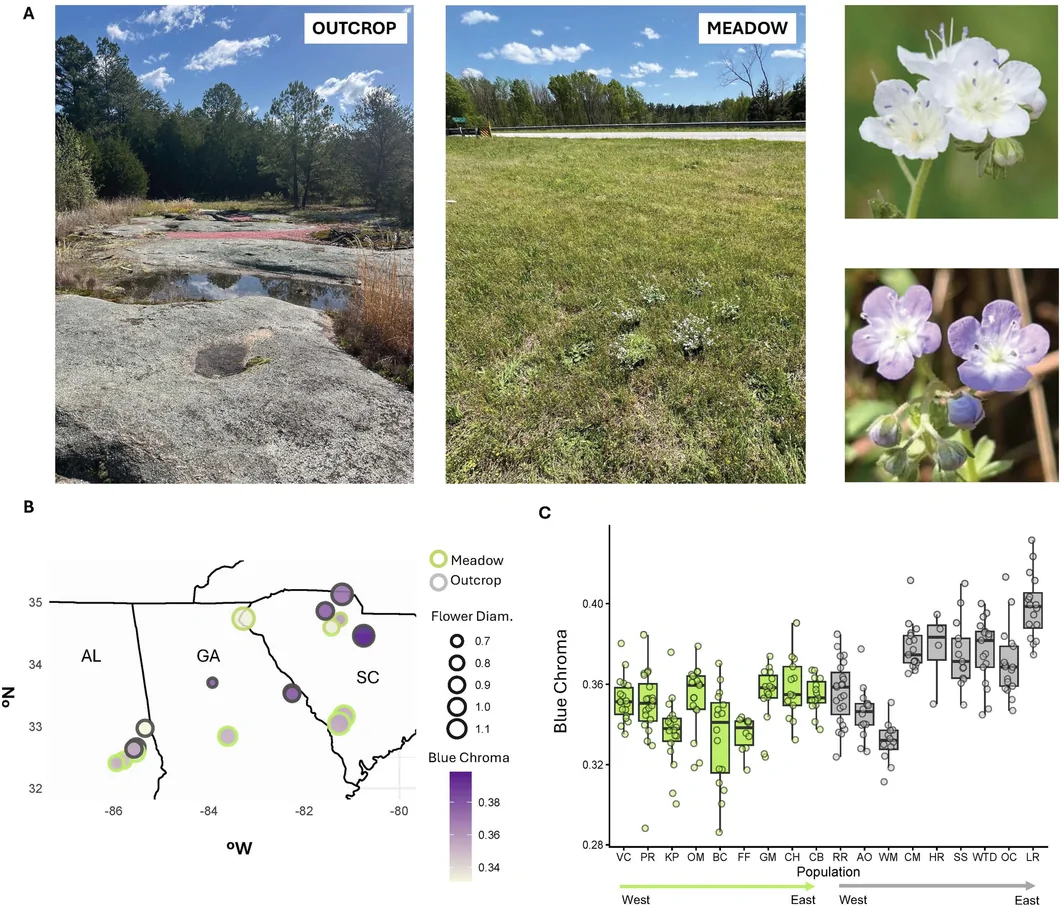
(A) Images of a natal outcrop and a recently colonized roadside meadow population of *Phacelia dubia* in the Southeastern United States, and images of flower color extremes. (B) A map of populations sampled in the field with points depicting habitat type, flower size, and petal blue chroma. (C) Box plots of petal blue chroma for each population. Within habitat type, populations are ordered from west to east to depict a longitudinal pattern of blue chroma for outcrop populations. Points represent flowers, boxplots represent the median, 25% and 75% quartiles, and the whiskers show are 1.5 × the interquartile range. Photos in panel (A) by M. Koski.

*Phacelia dubia* (Hydrophyllaceae) is a winter annual distributed east of the Mississippi River from Southern Alabama to Pennsylvania. It is common on the Appalachian and Piedmont Plateaus, and rare in the coastal plain. We focused this study on Piedmont populations in the Southeastern United States where it grows on the edges of vegetated moss and lichen mats in granite rock outcrops, and in grassy meadow‐like habitats on roadsides and lawns (Table S1). The first herbarium collections from roadside and lawn habitats occurred between 1972 and 1984 (Taylor‐Bennetts and Levy 2024), suggesting recent expansion from outcrops. In the Southeastern US Piedmont region, *P. dubia* is endemic to granite outcrops. To sample floral phenotypes and pollinators, we selected several meadow and granite outcrop populations in Alabama, Georgia, and South Carolina. Populations occupying the two distinct habitat types did not differ in average latitude or longitude (Figure 1B). Germination occurs in autumn; plants overwinter as a rosette and then flower from late February to May. One study in a Southwestern Virginia population documented flower visitation by syrphid flies, and a variety of polylectic bees, especially halictids (del Castillo 1994), though detailed profiling of the pollinator community has not been done.

#### 2.1.1 Floral Phenotypes in the Field

To quantify flower color variation within and among populations in the field we collected a single flower from up to 20 separate plants (*N* = 277) from 18 populations (mean per population = 15.4) at least 1.5 m from one another during peak flowering in Spring 2022. One population (FF) was discovered and sampled in 2023. Petal reflectance from the distal tip of one petal per flower was measured using an Ocean Optics spectrophotometer (Ocean Optics, Dunedin, FL, USA), with a deuterium‐tungsten UV–NIR light source placed at a 90° angle to capture reflectance of flower petals. Petals are not glossy, so diffuse reflection is likely minimal. Using the *pavo* package, reflectance curves were smoothed with a parameter of 0.25 between 300 and 700 nm. To quantify variation in petal color, we measured blue chroma as *R*_*λ*400–510_/*R*_*λ*300–700_. Blue chroma captures the most variation in petal reflectance of *P. dubia* because flowers lack UV reflectance (Figure S1). Visibly paler flowers have lower blue chroma while darker flowers have higher blue chroma, making it an intuitive measurement of color with respect to human‐perceived color. We photographed flowers in the field in Spring 2022 and 2023 with a ruler held at flower height and used digital image analysis in imageJ to extract the flower diameter of an average of 37.8 flowers per population (*N* = 860).

#### 2.1.2 Floral Phenotypes in the Greenhouse

In Spring and Summer 2023 we collected entire fruiting plants in separate envelopes from natural populations. In Fall 2023 we sowed between 3 and 6 field‐collected seeds per maternal family from 12 populations in Petri dishes (6 outcrops, 6 meadows; *N* = 168 maternal families). We also sowed 21 seeds from one outcrop population in Georgia that was collected such that seeds could not be assigned a maternal identity. Seeds were allowed to germinate at 15.5°C/14.5°C day/night with 12 h days in Percival growth chambers. Upon emergence of a radicle and cotyledons, seeds were transplanted to flats in germination mix kept in the same conditions as seeds. After about 2 weeks of rosette growth, we potted seedlings into conical pots in a 3:1 mix of Fafard to coarse sand and put them in a greenhouse set to 21°C/15.5°C day/night with 16 h daylengths. Germination rate was 40% on average. An average of 19.9 plants per population germinated and survived to flowering.

Upon flowering, we collected two flowers per plant and measured spectral reflectance from the adaxial petal tip in the same way we scored reflectance on field‐collected flowers. On two separate flowers per plant, we also measured flower diameter using digital calipers. In Fall 2024, we sowed seed from two additional meadow and two additional outcrop populations from which seed was collected in 2024, as well as additional seed from three populations with poor germination or survival from the 2023 common garden. Seeds, seedlings, and adult plants were treated in the same way as in 2023 and experienced the same greenhouse conditions. In total, we scored reflectance and flower diameter on 326 individuals from 16 populations. The number of plants per maternal family ranged from 1 to 6. From the two flowers scored per plant, we averaged blue chroma and flower diameter.

#### 2.1.3 Pollinator Collection and Identification

To characterize the composition of *P. dubia* 's pollinator community within each population, we collected insects directly from its flowers in 17 focal populations (Table S1) across the flowering seasons in Spring 2023, 2024, and 2025. In 2023 and 2024, we collected pollinators at each site one to three times per year and aimed to collect across various times during the day: morning (09:00–12:00), mid‐day (12:01–15:00), and evening (15:01–18:00). During each visit, we aimed to collect 30 insects or collect until 2 h had been reached. After two field seasons, we evaluated our sampling effort, and in 2025 we conducted targeted sampling in populations that were poorly sampled in previous years. Across years and sites, 35.4% of sampling events were conducted in the morning, 43.5% during mid‐day, and 24% in the evening. We compiled 1242 insects from 62 sampling events and pinned and identified them to family (mean = 73.05 insects/population) with the aid of a dissecting microscope. To identify Hymenopteran families, we primarily relied on wing venation patterns, the location of scopal hairs, and the presence/absence of facial fovea and rima (Michener 2007). When possible, we identified to genus and/or species; however, we restricted all analyses to the family level because of limited confidence of lower‐level identities of many Andrenidae, Megachilidae, Halictidae, and Fanniidae.

#### 2.1.4 Pollinator Efficiency

For the seven most abundant pollinator families across all populations (> 20 individuals collected), we estimated two proxies of pollinator efficiency—(1) *P. dubia* pollen carrying capacity and (2) fidelity to *P. dubia* . In order from highest to lowest abundance these were: Andrenidae, Syrphidae, Apidae, Megachilidae, Halictidae, Faniidae, and Buprestidae. We randomly selected between 4 and 22 individual insects per family from collections made in 2023 and 2024 (average = 8.14/family). Buprestidae was represented by only *Acmaeodera tubulus* and Faniidae was represented by only *Fannia* sp. Within families with more taxonomic diversity, the random sampling represented diversity within families (Table S2). For example, in the entire insect collection identified as Apidae, 66% were *Apis mellifera* , 22.5% were in the genus *Ceratina*, 8.3% were *Nomada*, and 2.5% were *Xylocopa*. Within the Apidae used for estimation of pollinator efficiency, five were *Apis mellifera* and one was a *Ceratina*.

To collect data on pollen carrying capacity and fidelity to *P. dubia* , we coated the tips of pins in melted fuchsin jelly which was allowed to then harden (“precision swab”; Donald et al. 2023). We gently swabbed the following areas on each Hymenopteran and Dipteran specimen, each with a separate swab: head (anterior), thorax (ventral), abdomen (ventral), anterior leg, mid leg, and posterior leg. Each swab was then melted onto its own glass microscope slide and sealed with a cover slip. For the one Coleopteran family (Buprestidae), we additionally swabbed the elytra. On each slide, we scored the number of *P. dubia* grains and the number of heterospecific grains. Grains scored as heterospecific differed in size and/or shape from *P. dubia* pollen, and/or possessed spines (Asteraceae). Heterospecific grains were not identified to species but simply lumped as “heterospecific.” We considered the total number of *P. dubia* grains to represent the pollen carrying capacity for a given insect, and the proportion of *P. dubia* grains to total grains ( *P. dubia* + heterospecific) to represent its fidelity to *P. dubia* . We multiplied pollen carrying capacity by fidelity as a metric of pollinator efficiency.

### 2.2 Data Analysis

#### 2.2.1 Floral Variation in the Field and Greenhouse

To test whether petal blue chroma and floral diameter measured in the field differed between habitat types, we modeled each as a function of habitat type with population nested in habitat type as a random term using a mixed effects linear model with a Gaussian distribution (*lmer*; lme4 package; Bates et al. 2015). We further assessed spatial patterns in floral traits using the same model structure with the inclusion of latitude, longitude, and their interactions with habitat type. When interactions were not significant, they were removed from the final model presented. We used “Anova” (car package; Fox and Weisberg 2019) with Type III sums of squares to test for significance of the fixed effect, and “ranova” (lmerTest package; Kuznetsova et al. 2017) to test for the significance of random terms.

For the greenhouse‐collected floral traits we tested for habitat differences using the same model described above with the inclusion of maternal identity nested in population identity as a random term. Then, we partitioned phenotypic variance among maternal identity and population using a random effects model with population and maternal identity nested in population as random effects. We used “ranova” to obtain sums of squares for each factor and divided sums of squares for each level by total sums of squares to determine the proportion of variance explained by each.

To assess the degree to which field average population trait values predicted average population trait values in the greenhouse, we regressed greenhouse population averages on field averages.

#### 2.2.2 Pollinator Efficiencies

To understand how the seven most common pollinator families varied in their efficiencies as pollinators of *P. dubia* we modeled the number of *P. dubia* pollen grains on each slide (our metric of pollen carrying capacity) as a function of pollinator family with insect identity and insect body part as random terms. Because pollen count data were zero‐inflated (i.e., several slides had zero *P. dubia* grains), we used a zero inflated Poisson distribution in the glmmTMB package (Brooks et al. 2017). We then modeled the proportion of *P. dubia* pollen to total pollen (our metric of fidelity to *P. dubia* ) with the same model but used a Gaussian distribution (*lmer*) because it was not zero‐inflated. From each model, we generated estimated marginal means (EMMs; Lenth and Piaskowski 2026) for each pollinator family and multiplied the EMMs as a metric of pollinator efficiency (e.g., if Apidae carried 120 *P. dubia* grains, and 80% of its total pollen load was *P. dubia* , its efficiency score was 120 × 0.80 = 96). We scaled the multiplicative efficiency scores by dividing each by the highest family‐level efficiency value which was Megachilidae.

#### 2.2.3 Association Between Pollinator Community, Habitat, and Flower Color

Flower diameter did not exhibit habitat‐dependent or spatial variation (see Section 3), and preliminary analyses showed it was unassociated with the pollinator community. Therefore, we focus results on associations between the pollinator community, habitat type, and flower color. We characterized pollinator communities by generating various iterations of pollinator community matrices at the population‐level—(1) an order‐level matrix including all pollinators collected, (2) a family‐level matrix subset to families represented by 10+ insects across all populations (*N* = 11 families; 91% of pollinators collected), and (3) a family matrix subset to families represented by 20+ insects in the collection (*N* = 7 families; 86.9% of all pollinators collected). Finally, we generated an efficiency‐weighted matrix of the most restrictive matrix (seven families). For each of these matrices, we partitioned variation in the pollinator community using nonmetric multidimensional scaling (NMDS) with Bray–Curtis dissimilarity (metaMDS; vegan package; Oksanen et al. 2026). We increased the number of dimensions to 3 for the family‐level matrices to reduce stress values to < 0.1, and plotted populations in NMDS space denoted by habitat type and color (white vs. purple). The cutoff for “purple” was an average population blue chroma > 0.37 which was represented by six outcrop populations sampled in the field (Figure 1C), five of which were sampled for pollinators (Table S1). We ran Analyses of Similarity (ANOSIM) using each matrix to test if pollinator communities differed significantly between habitat types and between populations with white versus purple flower color with 9999 permutations and Bray–Curtis Distance. Because flower color variation is quantitative in nature, we further tested for correlations between average population blue chroma and NMDS axes. We did the same for flower diameter.

We then tested whether pollinator community differences were associated with blue chroma color differences between populations. We generated a Euclidean distance matrix for blue chroma between each population and tested whether it correlated with the Bray–Curtis pollinator community distance matrix using each of the three family‐level pollinator community datasets—at least 10 insects, at least 20 insects, and the efficiency‐weighted matrix. We tested for correlations using only meadow‐meadow pairwise comparisons, only outcrop‐outcrop comparisons, and only outcrop‐meadow comparisons to determine if pollinator community differences exacerbated color differences when associated with habitat type differences.

Finally, we assigned each population to a pollination module following Moir et al. (2025) using the most restrictive family‐level matrix (20+ insects/family) and the efficiency‐weighted matrix. First, we generated bipartite networks with pollinator families and populations as nodes to visualize relationships between flower color and the pollinator community (bipartite package; Dormann et al. 2009). We then computed pollination modules using the “Beckett” method (Beckett 2016) and evaluated how habitat type and population‐level flower color clustered within and between modules. Modules indicate populations that group together based on a similar pollinator community. We tested whether modules differed significantly in flower color by modeling blue chroma from flowers in the field as a function of module identity with population as a random term (*lmer*).

#### 2.2.4 Testing Associations Between Flower Color and Abundant Versus Efficient Pollinators

For the seven most dominant pollinator families we regressed population‐average blue chroma from the field on the proportional representation of each family in a population. This yielded an *R* ^2^ value for each pollinator family (Figure S3). For example, the proportion of Halictidae within populations explained ~10% of the variation in flower color among populations. We then regressed the proportion of flower color variance explained by each pollinator family on the abundance of that family in the entire dataset. We predicted that if the most abundant pollinators explain the most variation in color, then there should be a positive relationship between pollinator abundance and variance explained. Then, we regressed the amount of color variance explained by each family on the efficiency of each family. If the most efficient pollinators explain the most variation in color, then there should be a positive relationship between pollinator efficiency and variance explained.

## 3 Results

### 3.1 Floral Phenotypic Variation in the Field

Visible flower color ranged from white (lower blue chroma) to deep purple (higher blue chroma) (Figure 1A) and showed minimal to no ultraviolet reflectance (Figure S1). On average, blue chroma was higher in outcrop populations (*F*_1,15.9_ = 7.35; *p* = 0.015), and there was significant variation among populations within habitat type (LRT = 115; *p* < 0.0001) (Figure 4B,C). Outcrop populations exhibited more among‐population variance for blue chroma than meadows (outcrop coefficient of variation = 0.053; meadow CV = 0.028). A model partitioning blue chroma variation that included latitude and longitude revealed a stronger habitat effect on blue chroma (*F*_1,12.7_ = 7.35; *p* = 0.006) than a model that did not account for space, and a significant Habitat × Longitude interaction (*F*_1,10.3_ = 7.35; *p* = 0.007). Outcrop flowers were lighter in the west and darker in the east, but there was no longitudinal influence on blue chroma for meadow populations (Figure 1C). There was a weak positive effect of latitude on blue chroma (*F*_1,13.0_ = 3.5; *p* = 0.08). There was no effect of habitat or space on flower diameter (all *p* > 0.23), though there was significant population‐level variation within habitat type (Figure 1B; *p* < 0.0001). Population‐average diameter and blue chroma were uncorrelated (*r* = −0.04, *p* = 0.87).

### 3.2 Floral Phenotypic Variation in a Common Garden

**FIGURE 2:**
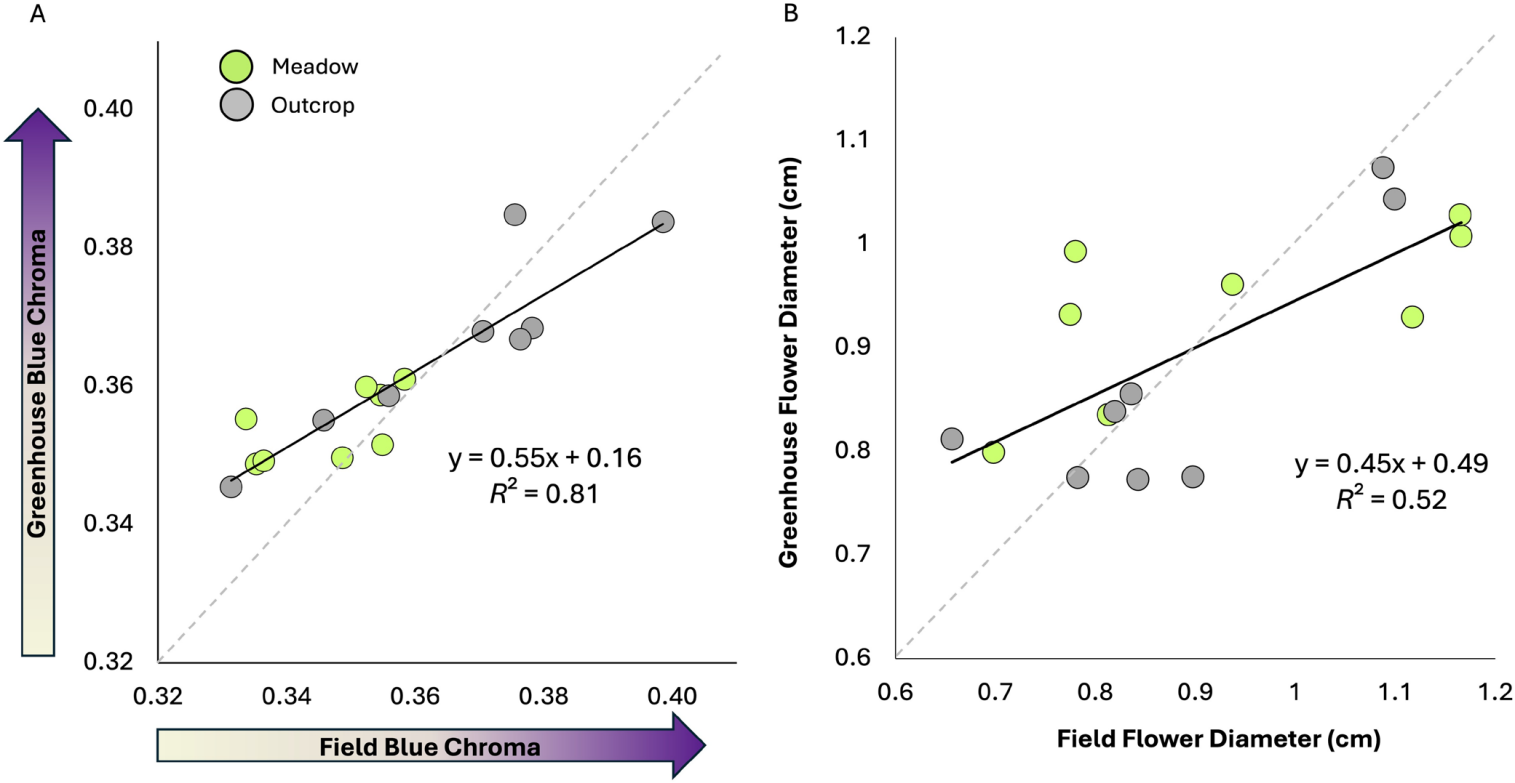
(A) Average population petal blue chroma from field‐collected flowers predicts population average blue chroma in the greenhouse. (B) Average population petal diameter measured of field collected flowers predicts population average petal diameter in the greenhouse. Dashed lines represent 1:1 relationships.

From field collected seed grown in the greenhouse there was an effect of habitat type on blue chroma (*F*_1,14.75_ = 7.52; *p* = 0.015), and significant variance explained by population within habitat type (LRT = 57.7; *p* < 0.0001), as well as maternal genotypes within population (LRT = 15.54; *p* < 0.0001). A random‐effects model including population and maternal identity revealed 55% of the variation in blue chroma was attributable to population identity and 10.7% was attributable to maternal identity within populations. Population level blue chroma in the field explained > 80% of the variation in the greenhouse (*t* = 7.6, *F*_1,14_ = 56.2; *p* < 0.0001; Figure 2A).

Flower diameter did not differ between habitat types (*F*_1,14.77_ = 1.43; *p* = 0.25) but differed significantly among populations within habitats (LRT = 85.2; *p* < 0.0001) and exhibited modest maternal genotypic variation (LRT = 3.37; *p* = 0.066). 42% of the variation in flower diameter was attributed to population identity while only 6.4% was attributable to maternal genotype within population. Average population diameter in the field predicted > 52% of the variation in the greenhouse (*t* = 3.94, *F*_1,14_ = 15.5; *p* = 0.001; Figure 2B). As was observed in the field, blue chroma and diameter were uncorrelated at the population level in the greenhouse (*r* = −0.04, *p* = 0.88).

### 3.3 Order‐Level Pollinator Community

**FIGURE 3:**
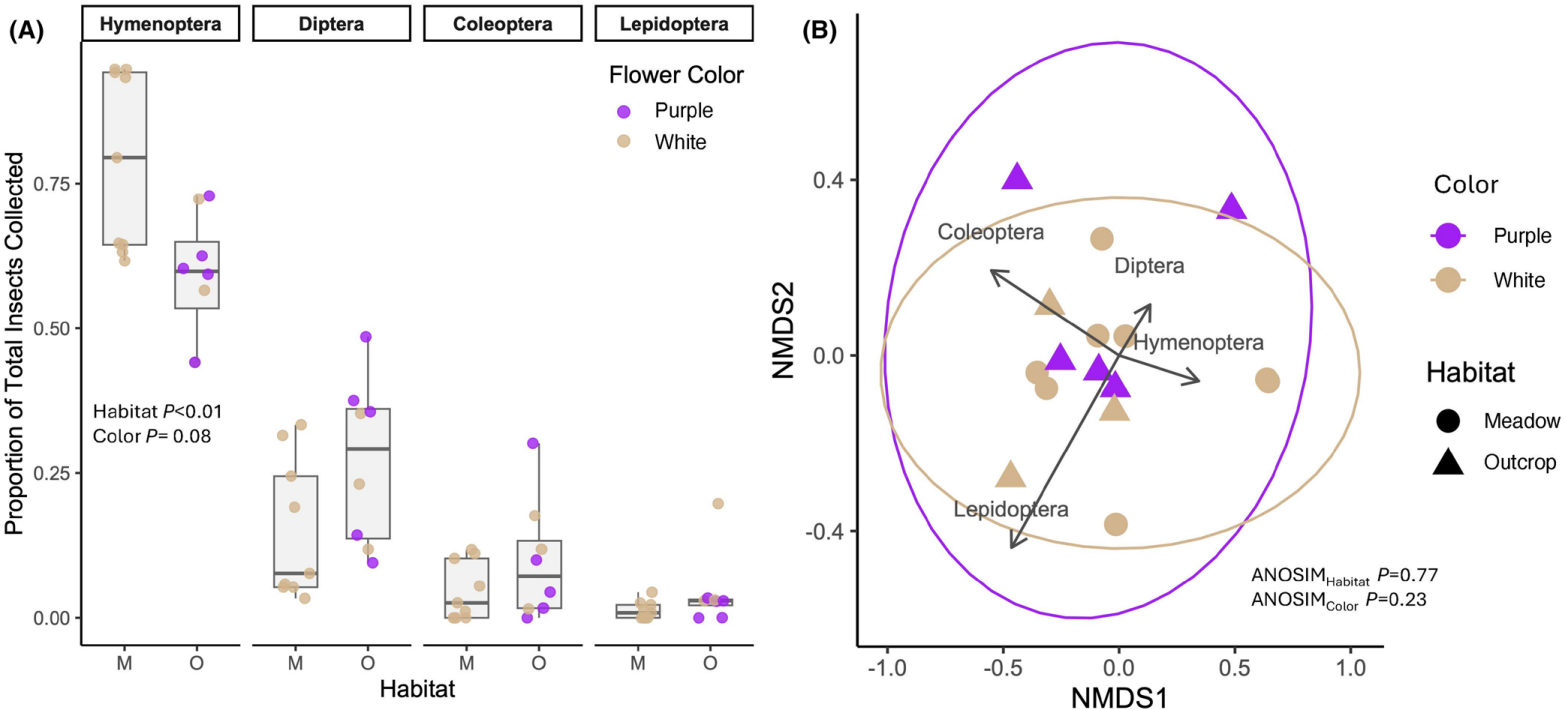
(A) The proportion of Hymenoptera, Diptera, Coleoptera, and Lepidoptera visiting *Phacelia dubia* flowers in meadow (M), and outcrop populations (O). Each point is a population colored by its binary flower color score (purple or white). Points represent populations, boxplots represent the median, 25% and 75% quartiles, and the whiskers show are 1.5 × the interquartile range. (B) NMDS plot of order‐level pollinator community of *P. dubia* populations with color depicting flower color and shape depicting edaphic habitat.

Across populations, Hymenoptera dominated the pollination community of *P. dubia* (71% of pollinators collected), followed by Diptera (20.25%), Coleoptera (6.15%), and Lepidoptera (2.6%). Hymenoptera had a higher relative abundance in meadow populations but did not differ between white‐ and purple‐flowered populations (Figure 3A). NMDS did not reveal differences in order‐level pollinator communities between habitat types or populations dominated by purple (mean blue chroma > 0.37) versus white (blue chroma < 0.36) flowers (ANOSIM_Habitat_ *p* = 0.77; ANOSIM_Color_ *p* = 0.23). NMDS scores and quantitative variation in blue chroma were uncorrelated (NMDS1 *r* = −0.03, *p* = 0.91; NMDS2 *r* = 0.32, *p* = 0.21). Moreover, there was no correlation between the proportion of Hymenoptera and blue chroma (*r* = −0.17; *p* = 0.52). There were weakly positive correlations between the proportion of Hymenoptera and flower diameter (*r* = 0.42; *p* = 0.09) and NMDS 1 and diameter (*r* = 0.48, *p* = 0.051).

### 3.4 Family‐Level Pollinator Efficiency

The number of *P. dubia* grains (*χ* ^2^ = 54.9; *p* < 0.0001; Figure S2A) and proportion of *P. dubia* grains relative to all grains (*F*_6,58.4_; *p* = 0.027; Figure S2B) on pollinator bodies differed among the seven most common pollinator families. Megachilidae and Apidae had significantly higher conspecific pollen loads than all families (all *p* < 0.05) but did not differ from one another. Apidae had a higher proportion of conspecific pollen than Syrphidae (*p* = 0.046) and as did Megachilidae though the significance was only marginal (*p* = 0.055). All other pairwise post hoc comparisons among families for the proportion of conspecific pollen did not differ. Combining the number of *P. dubia* grains and proportion of conspecific grains into a metric of pollination efficiency, there was a strong family‐level difference (*χ* ^2^ = 54.9; *p* < 0.0001; Figure S2C). Megachilidae carried the most *P. dubia* grains on average (182.6 ± 95.4 SE; Figure S2A) and carried almost exclusively *P. dubia* pollen (0.936 ± −0.06 SE; Figure S2B). Apidae (all *Apis mellifera* ), had the second highest pollen carrying capacity and strong fidelity to *P. dubia* (131.7 ± 77 SE; *P. dubia* grains; 96.4% conspecific; Figure S2A,B). Megachilidae had significantly higher efficiency than all other groups (all *p* < 0.05) except Apidae (*p* = 0.68) (Figure S2). Apidae had higher efficiency than all groups except Andrenidae (*p* = 0.11) (Figure S2).

### 3.5 Family‐Level Pollinator Community

**FIGURE 4:**
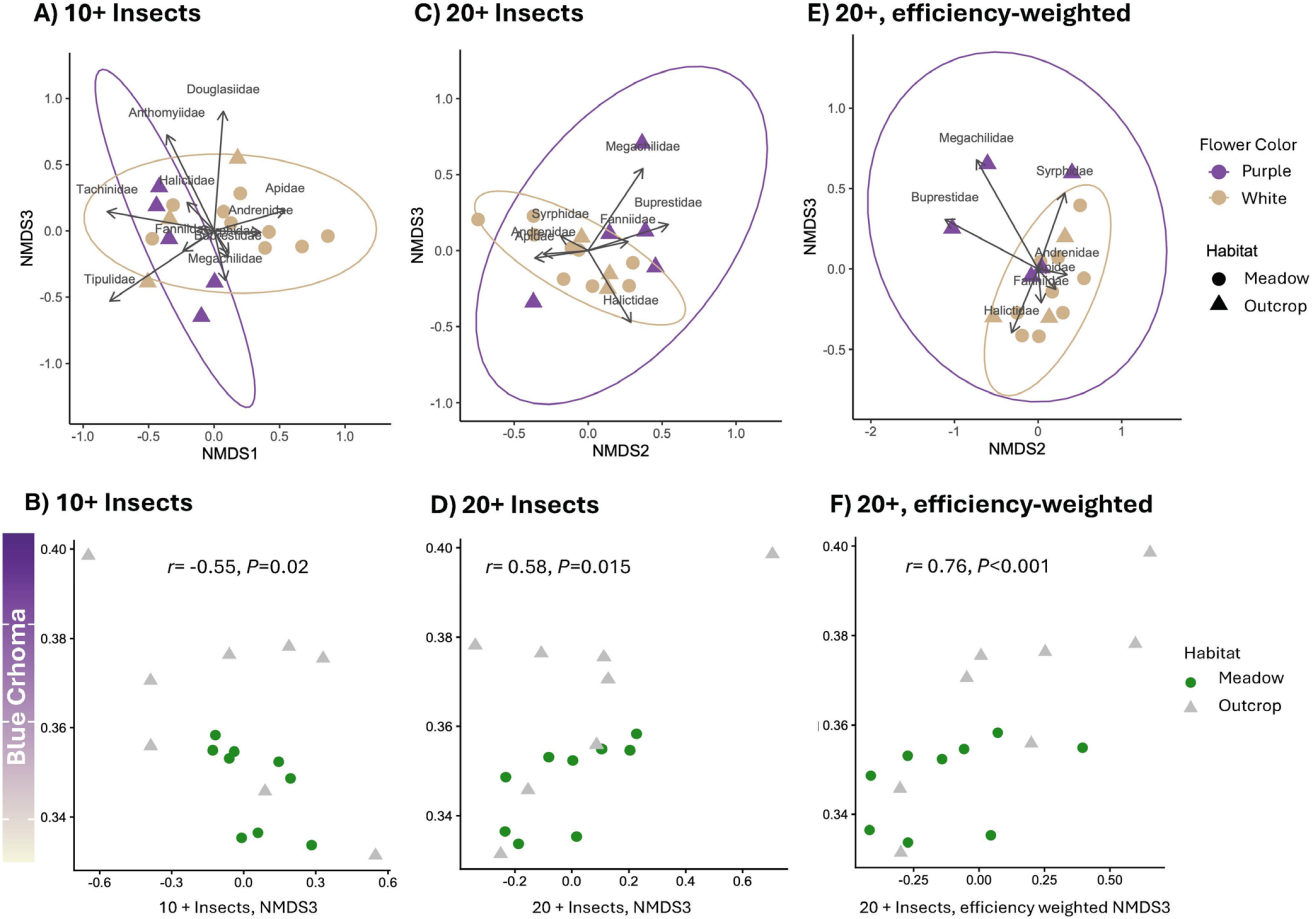
NMDS plots of pollinator communities based on family‐level insect classification (panels A, C, E) with each population depicted by flower color and by edaphic habitat. Purple and beige circles represent 95% CI ellipses for purple and white‐flowered populations, respectively. Arrows and associated insect families represent loadings on NMDS axes. The NMDS axes plotted were chosen to depict the primary differences between groups. (A) NMDS plot using all insect families with at least 10 insects collected across all populations (*N* = 11 families). (C) NMDS plot using all insect families with at least 20 insects collected across all populations (*N* = 7 families). (E) NMDS plot using families with at least 20 insects collected, weighted by pollinator efficiency. Panels (B), (D), and (F) show significant relationships between NMDS axes for each community matrix and quantitative variation in blue chroma among populations.

Using the pollinator community matrix including only families with ≥ 10 insects collected across all populations (*N* = 11 families; 91% of collection), there were significant differences between habitat types, and between dark and light‐flowered populations (Figure 4A; Table 1). Lighter‐flowered meadow populations tended to have more Apidae and Andrenidae (Figure 4A). NMDS3, on which Megachilidae and Tipulidae loaded negatively (Figure 4A), was negatively correlated with quantitative variation in blue chroma (Figure 4B). Other NMDS axes did not correlate with blue chroma, and none correlated with flower size (all *p* > 0.05).

**TABLE 1:** Results from Analysis of Similarity (ANOSIM) comparing pollinator communities between edaphic habitats (meadow vs. outcrop) and dominant flower color (white vs. purple) of *Phacelia dubia* populations.

Pollinator community matrix	Habitat ANOSIM		Color ANOSIM	
	*R*	*p*	*R*	*p*
Order level	0.044	0.234	−0.120	0.774
Family level, 10+ insects	0.211	0.008	0.278	0.053
Family level, 20+ insects	0.192	0.015	0.262	0.058
Family level 20+, efficiency‐weighted	0.254	0.003	0.373	0.020

*Note:* *R* values closer to 1 indicate stronger pollinator community composition dissimilarity between groups, while those closer to 0 indicate populations do not cluster based on groupings.

Further sub‐setting to families with ≥ 20 insects collected (*N* = 7 families; 86.9% of collection), NMDS again revealed differences in pollination communities between habitat types and between dark and light‐flowered populations (Figure 4C; Table 1). NMDS 3, on which Megachilidae loaded positively and Halictidae loaded negatively (Figure 4C), was positively associated with quantitative variation in blue chroma (Figure 4D). Other NMDS axes did not correlate with blue chroma, and none correlated with flower size (all *p* > 0.05).

Finally, the pollinator matrix weighted by pollinator efficiency yielded the strongest pollinator community differences between habitats and between light and dark‐flowered populations (highest *R*‐values, lowest *p*‐values; Table 1; Figure 4E). Moreover, NMDS3 from the efficiency weighted matrix was tightly positively correlated with blue chroma (Figure 4F). Megachilidae, Syrphidae, and Buprestidae loaded positively on NMDS3 (Figure 4E). Other NMDS axes did not correlate with blue chroma, and none correlated with flower size (all *p* > 0.05).

### 3.6 Relationship Between Flower Color and Pollinator Community Distance

**FIGURE 5:**
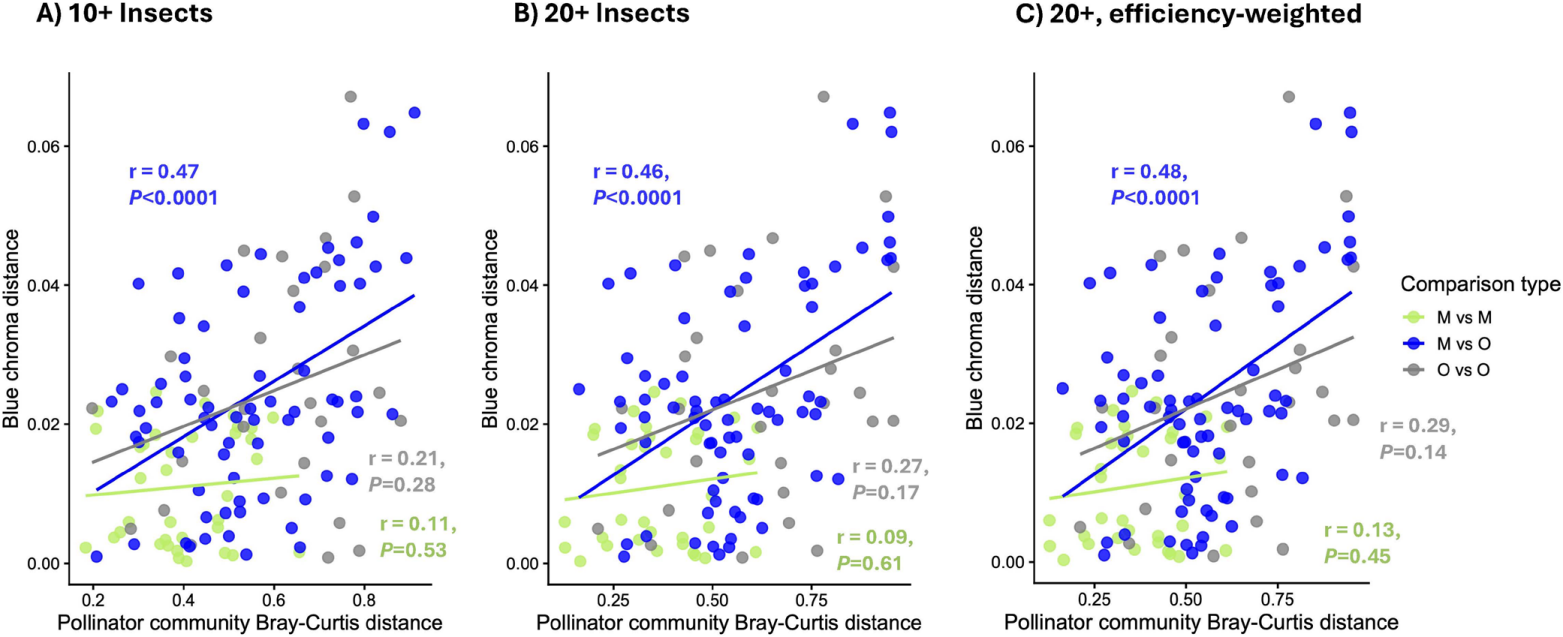
Pairwise distance between pollinator communities predicts flower color divergence between habitat types. Pairwise Euclidean blue chroma distance is plotted against Bray–Curtis pollinator community distances for the pollinator community matrix including all insect families with at least 10 insects (A), at least 20 insects (B), and an efficiency weighted matrix (C). Meadow‐meadow (M vs. M), outcrop‐outcrop (O vs. O), and meadow‐outcrop (M vs. O) comparisons are depicted by different colors and are associated with Pearson correlations.

Pairwise pollinator community distance between populations was positively correlated with pairwise blue chroma distance using each of the pollinator community matrices (all *r* > 0.53, *p* < 0.0001; Figure 5). The overall correlation was driven by meadow‐outcrop comparisons (all *r* > 0.46, *p* < 0.000) and to a lesser extent outcrop‐outcrop comparisons. Outcrop‐outcrop comparisons yielded positive relationships, but none were significant (all *p* > 0.13; Figure 5). Pollinator community differences between meadow populations were lower than other comparisons and were not associated with color differences (Figure 5).

### 3.7 Family‐Level Pollination Modules

**FIGURE 6:**
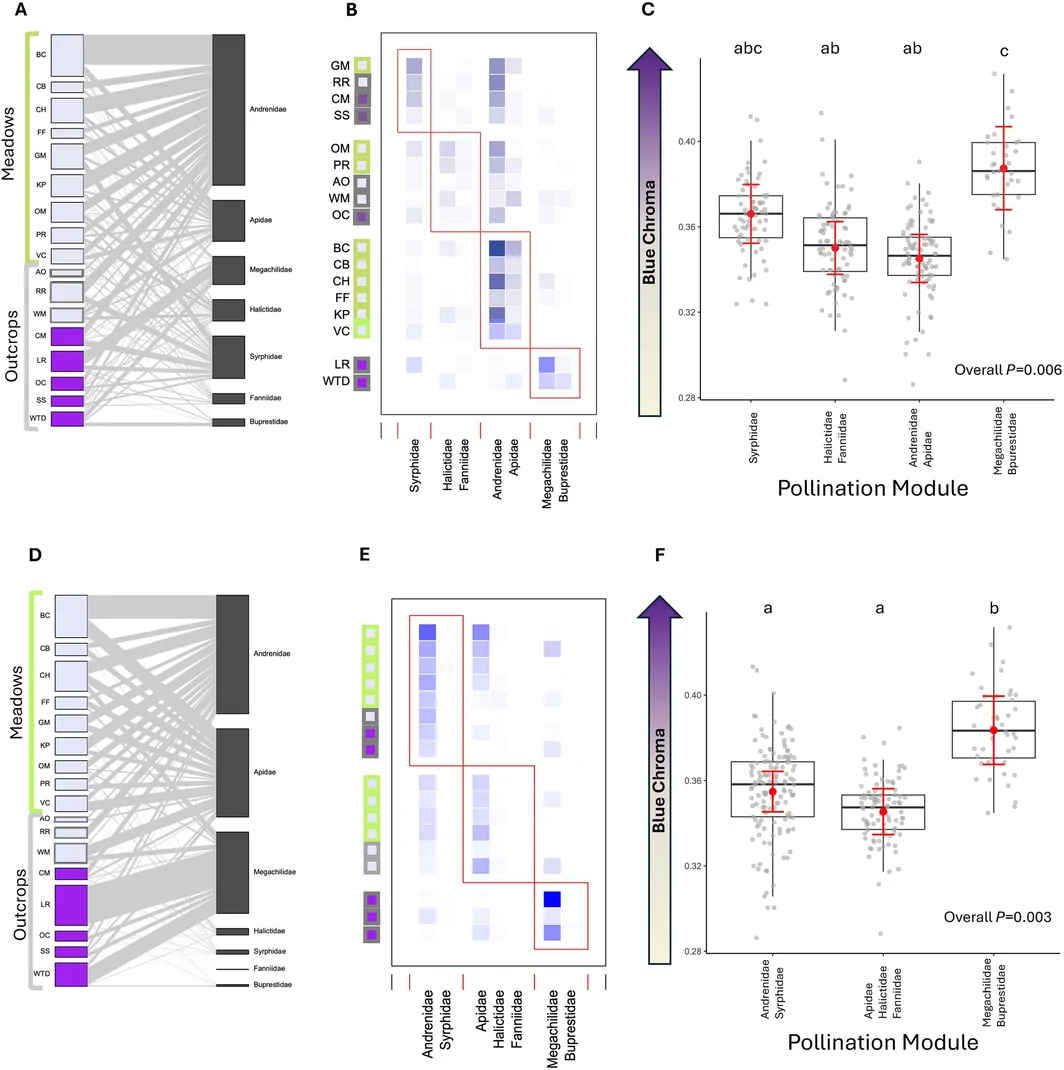
(A) The abundance‐based bipartite network between *Phacelia dubia* populations (left) and the seven most common pollinator families. The width of gray lines indicates interaction strength. (B) Pollination modules based on a clustering algorithm using the bipartite network in panel (A). Meadow populations are outlined in green and outcrops in gray, and the interior color depicts flower color. Darker blue squares indicate higher abundance of a given pollinator within a population. (C) Petal blue chroma for each pollination module. Points represent individual flowers, boxplots represent the median, 25% and 75% quartiles, and whiskers are 1.5 × the interquartile range. Red points and error bars are estimated marginal means and 95% CIs from a mixed model with pollination module as a fixed term and population as a random term. (D) The bipartite module based on a pollinator‐efficiency weighted community matrix, the resultant pollination modules (E), and petal blue chroma for each pollination module (F). Modules that do not share a lowercase letter in panels (C) and (F) are significantly different from one another based on post hoc Tukey tests.

The family‐level pollinator community matrix unweighted by pollinator efficiencies recovered four pollinator modules—Module 1: Syrphidae; Module 2: Halictidae and Fanniidae; Module 3: Andrenidae and Apidae; and Module 4: Megachilidae and Buprestidae (Figure 6A,B). The Andrenidae/Apidae module was comprised of six meadow populations, and the Megachilidae/Buprestidae module was comprised of two purple‐flowered outcrops. The Syrphidae and the Halictidae/Fanniidae modules were both comprised of a mix of populations defined by habitat and color. The Megachilidae/Buprestidae module had significantly higher blue chroma than the Halictidae/Fanniidae and Andrenidae/Apidae modules (Figure 6C). Blue chroma in the Syrphidae module did not differ from the Megachilidae/Buprestidae module (Figure 6C). Flower size did not differ among modules (*F* = 0.11, *p* = 0.96).

The efficiency‐weighted pollinator matrix recovered three modules—Module 1: Andrenidae and Syrphidae; Module 2: Apidae, Halictidae, and Fanniidae; and Module 3: Megachilidae and Buprestidae (Figure 6D,E). The Apidae/Halictidae/Fanniidae module, defined largely by high Apidae interaction, was comprised of six white‐flowered populations from meadows and outcrops. The Megachilidae/Buprestidae module was comprised of three purple‐flowered outcrops. The Andrenidae/Syrphidae module contained all types of populations. The Megachilidae/Buprestidae module had significantly higher blue chroma than either of the other two modules (Figure 6F). Again, flower size was unrelated to module.

### 3.8 Does Variation in Abundant or Efficient Pollinators Better Explain Color Variation?

**FIGURE 7:**
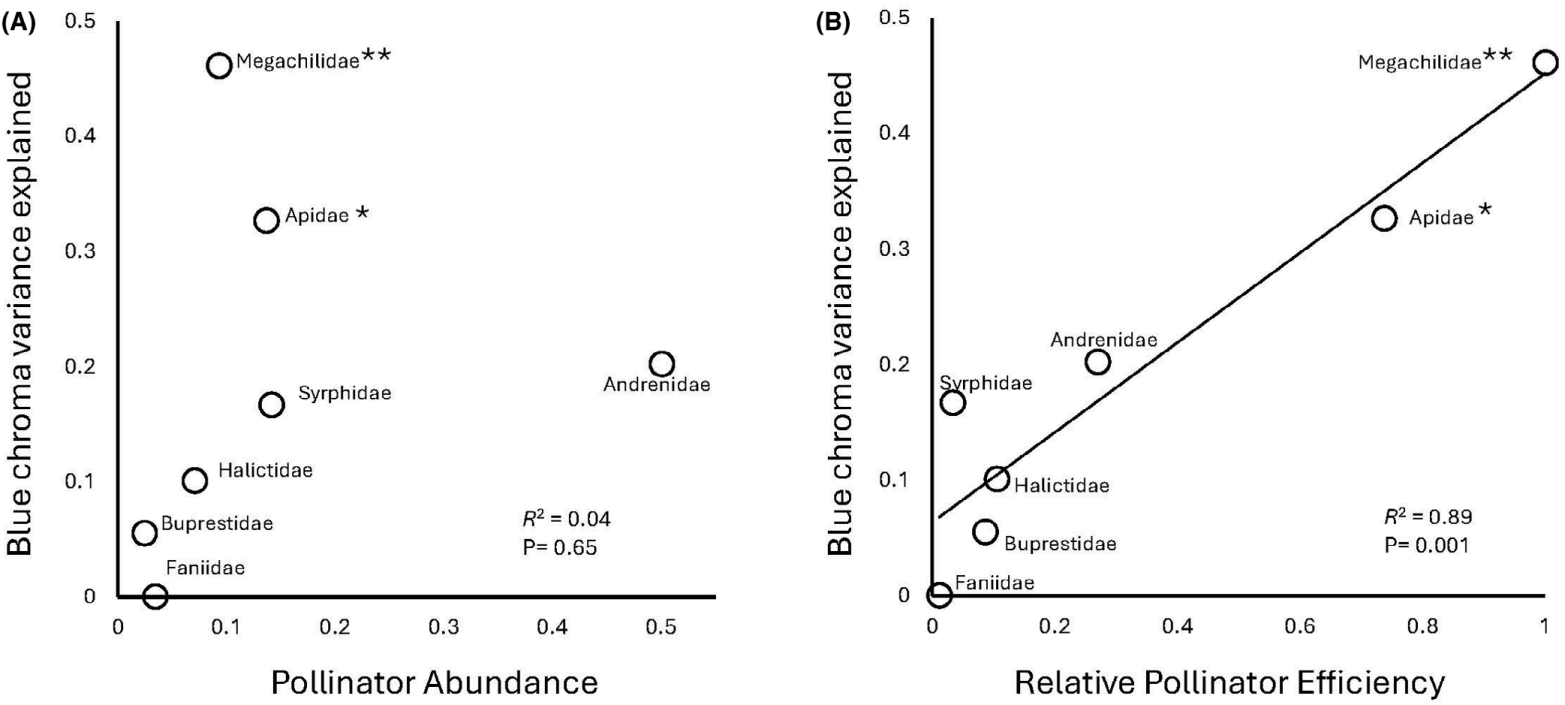
(A) The relationship between the relative abundance of the seven most common *P. dubia* pollinators pooling across all populations, and the proportion of among‐population variance in blue chroma explained by variation in relative abundance of each pollinator among populations. (B) The relative efficiency of the seven most common pollinator families of *P. dubia* , and the proportion of among‐population variance in blue chroma explained by variation in relative abundance of each pollinator among populations. In each panel asterisks indicate the following: *variation in pollinator abundance is significantly associated with variance in blue chroma at *p* < 0.05; **variation in pollinator abundance significantly associated with variance in blue chroma at *p* < 0.001.

Flower blue chroma significantly increased with the proportion of Megachilidae (*R* ^2^ = 0.46, *p* < 0.01) but decreased with the proportion of Apidae (*R* ^2^ = 0.33, *p* < 0.05) across populations (Figure S3). Two populations with high Megachilidae relative abundance (> 40%) contributed strongly to the covariance between blue chroma and Megachilid abundance but relative abundance in Apidae showed more continuous covariance with blue chroma (Figure S3). There was a tendency for higher blue chroma in populations with more Syrphid visitation (*p* = 0.10) and lower blue chroma in populations with more Andrenidae (*p* = 0.07) (Figure S3). The variance in blue chroma among population explained by the relative abundance of a given family was unassociated with the family's abundance in the entire pollinator collection (Figure 7A) but was strongly positively associated with its pollination efficiency (Figure 7B). Andrenidae was the most common pollinator across populations, and most common within 14 of 17 populations, but explained less variation than Megachildae and Apidae (Figure 7) which were more efficient in terms of pollen carrying capacity and fidelity to *P. dubia* .

## 4 Discussion

Our study describes patterns of floral variation in natal and recently colonized weedy populations of *Phacelia dubia* and assesses the potential for heterogeneity in pollinator mutualisms to shape it. Natal outcrop populations had darker flowers on average and exhibited a wider range of color than meadows. Coupled with a strong genetic underpinning for color, this suggests that outcrops harbor more genetic variance for petal color than meadows. *Phacelia dubia* 's pollinator community differed between edaphic habitats. Meadows were dominated by Andrenid bees and *Apis mellifera* , while outcrops exhibited wider variation in pollinator communities, with several characterized by Megachilid bee and Buprestid beetle visitation which were rare in meadows. Pollinator community differences were positively correlated with flower color differences between populations, especially for outcrop‐meadow comparisons, suggesting that habitat differences combined with pollinator differences spur stronger floral divergence, supporting van der Niet et al. (2006). There were correlations between flower color and the relative abundance of the two most efficient pollinator families (Megachilidae, Apidae). Spatial variation in these two families predicted more phenotypic variation than more abundant but less effective families (Andrenidae, Syrphidae). While several ecological differences between edaphic habitats may contribute to floral diversity, our study found support for a contribution from pollinator community composition. It also underscores the importance of the most effective pollinator principle when testing for signatures of the Grant–Stebbins model of floral evolution.

### 4.1 The Role of Pollinator Community Composition in Shaping Phenotypic Variation

While several non‐mutually exclusive processes may result in phenotypic evolution during the colonization and establishment process, we evaluated the role of novel selection regimes experienced following colonization. Our study addressed this scenario by relating floral variation to the pollination community, a key driver of selection on floral phenotypes. The reduced variance in flower color among meadow populations, and lighter flower color on average, could be driven in part by differences in the pollinator community available to *P. dubia* between habitat types. The family‐level pollinator community was more uniform across meadow populations than outcrop populations (Figure 3). This uniformity may contribute to the reduction in color variance among meadows assuming similar pollinator communities between populations results in similar selection on flower color. Moreover, a higher relative abundance of both Apidae and Andrenidae was linked with lighter flowers across all populations (Figure S3). Meadow populations largely fall within a pollination module characterized by either Apidae or Andrenidae, especially when modules are calculated based on efficiency‐based metrics (Figure 5E). This suggests that these two pollinator groups should be the primary agents of selection on floral phenotypes in meadows. Because extreme purple floral phenotypes do not exist naturally in meadows (Figure 1), an experimental approach will be required to test whether pollinators indeed prefer white flowers in meadow populations.

Outcrop flowers were darker on average compared to meadow flowers, but exhibited a strong longitudinal cline in flower color—lighter in the west, darker in the east—which is suggestive of geographic variation in selection on color. The results suggest that pollinator‐mediated selection could shape this geographic cline. Post hoc analyses of longitudinal variation for the seven dominant pollinator families of *P. dubia* using only outcrops revealed a strong increase in the relative abundance of Megachilidae from western to eastern outcrops (*b* = 0.08 ± 0.03 SE; *p* = 0.04, *R* ^2^ = 0.53). The other six dominant pollinators showed no longitudinal patterns. This suggests a primary role of Megachilidae in shaping selection on flower color. Megachilidae have exhibited preference for purple pollen over white pollen in a pollen color polymorphic species (Ison et al. 2019) and *Osmia lignaria* (Megachilidae) exhibits innate preference for artificial blue flowers over white flowers (Amaya‐Márquez et al. 2008). To date, however, behavioral assessments of color preferences of Megachilidae have not been conducted using *P. dubia* .

Our study highlights the importance of measuring pollinator efficiencies for generalist pollinated plants when assessing signatures of the Grant–Stebbins model of floral evolution. Most studies link floral variation with observations of pollinator frequency across space (e.g., Gómez et al. 2009; Wenzell et al. 2023) while few consider pollinator efficiencies (however see Moir et al. 2025). In our study, considering pollinator efficiencies enhanced the power of pollinator community data for explaining variation in floral traits among populations using both NMDS analyses and pollination module analyses. Furthermore, the variance in color explained by very abundant but less inefficient pollinators like Andrenidae and Syrphidae was low compared to highly efficient Megachilidae and Apidae. Geographic variance in the relative abundance of bees with different pollen deposition potential drives geographic variation in pollen limitation in *Campanula americana* (Koski et al. 2018), and thus the opportunity for selection on floral traits (Emel et al. 2017). To our knowledge, however, this is the first study to link pollinator efficiency with the proportion of phenotypic variance explained by pollinators relative abundance in populations.

An outstanding question that emerges from this study is: What drives variation in pollinator communities between habitat types and among populations? First, the coflowering community of ruderal grassy meadows likely differs from granite rock outcrops. Granite outcrops in the southeast are notable for high flowering diversity and high endemism (Wyatt and Allison 2000). Different flowering plant composition is known to impact the composition of pollinator communities available to a given species (Potts et al. 2003). Second, anecdotal evidence suggests that meadow populations flower earlier than outcrop populations (Taylor‐Bennetts and Levy 2024). Phenological differences in the emergence times of various pollinator groups could contribute to pollinator community differences (Rafferty and Ives 2012). We are currently assessing the contributions of the coflowering community and phenology of *P. dubia* in shaping differences in pollinator communities.

### 4.2 Other Processes Shaping Floral Phenotypic Variation

Historical demographic processes can contribute to geographic patterns of floral color diversity (e.g., Koski and Galloway 2020). Recently colonized populations may harbor a subset of the genetic variance for floral phenotypes compared to source populations, altering the phenotypic distribution in the new populations. First, our data support a reduction in genetic variance for flower color due to drift experienced during recent founder events. Second, the lighter floral color on average in meadows may suggest that they were primarily founded by outcrop sources with lighter flowers. While that data at hand cannot rule out the second scenario, it is unlikely given that weedy populations are most likely founded by nearby outcrops, and white‐flowered meadows are very near purple‐flowered outcrops in South Carolina and Georgia (Figure 1B). A population genetic analysis is currently underway to evaluate the roles of historical processes in shaping flower color variation.

The signaling habitat has the potential to shape pollinator perception of floral signals like color, and thus pollinator preferences (Koski 2020; Finnell and Koski 2021). Flowers in rock outcrops and meadows signal against very different backgrounds, and background color has a strong influence on pollinator‐perceived floral color (Bukovac et al. 2017). Because bees use their long‐wavelength (green) photoreceptor from a distance to distinguish flowers from the background (Giurfa et al. 1996), the green meadow background and grayer outcrop background likely influence floral conspicuousness. Ongoing work in this system is evaluating how variation in background color shapes pollinator‐perceived color. While a model of sensory drive for floral evolution posits that floral signal divergence may occur in the absence of pollinator shifts (Koski 2020), it does not exclude the potential for both pollinator shifts and differences in signaling habitats to jointly shape floral evolution.

Finally, non‐pollinator agents of selection on flower color are common (Strauss and Whittall 2006). Selection by the thermal environment (Koski and Galloway 2018; Lacey 2026), soil moisture (Warren and Mackenzie 2001), or light (Koski and Ashman 2015; Koski et al. 2022) may act directly on flower pigmentation. Alternatively, selection may act on the anthocyanin pigment pathway in vegetative tissue such that it impacts flower color (Armbruster 2002; Wessinger and Rausher 2012). Signatures of abiotic selection on plant coloration often result in predictable geographic clines (e.g., latitude/longitude) that covary with abiotic factors. There was a longitudinal cline in flower color in outcrops only, despite meadows spanning a similar longitudinal spread. Outcrops may present plants with extreme conditions like high day time heat, and reduced soil moisture due to thin soil layers. So, if there are strong longitudinal clines in abiotic factors that impact floral color, meadow populations may be buffered from abiotic stress relative to outcrop populations, which could result in reduced phenotypic differentiation.

## 5 Conclusions

Results show that weedy roadside and lawn populations of a granite rock outcrop endemic exhibit reduced flower color variation and have largely lost darker floral morphs that are common in natal outcrops. Meadow populations also experience reduced geographic heterogeneity in pollination communities relative to outcrops and a higher frequency of Apidae and Andrenidae, pointing to a role of novel pollination communities following colonization in shaping floral phenotypic evolution. This work additionally stresses the importance of quantifying metrics of pollinator efficiency, especially for generalist insect‐pollinated flowers. The relative frequency of bees with both high pollen‐carrying capacity and strong fidelity to the focal plant species predicted variation in flower color very well. Conversely, geographic variation in very common flower visitors that exhibited lower pollination efficiency was poor at predicting floral variation. Thus, as plants experience novel pollination communities following expansions and introductions, it is critical to examine not only the presence but the effectiveness of novel pollinators.

## Author Contributions

**Katherine E. Riddle:** data curation (lead), formal analysis (supporting), investigation (equal), writing – original draft (supporting). **Taylor N. Sherer:** data curation (equal), investigation (equal). **Jenna McCarty:** data curation (equal), investigation (equal), methodology (equal). **Stacy Taylor‐Bennetts:** investigation (supporting). **Matthew H. Koski:** conceptualization (equal), data curation (equal), formal analysis (equal), funding acquisition (equal), investigation (equal), methodology (equal), project administration (lead), resources (lead), supervision (lead), validation (lead), visualization (lead), writing – original draft (lead), writing – review and editing (lead).

## Funding

This work was supported by Division of Environmental Biology (2237529).

## Conflicts of Interest

The authors declare no conflicts of interest.

## Supporting information


**Table S1:** Location and data collected for focal populations.
**Table S2:** Pollinators used for pollination efficiency metrics.
**Figure S1:** Average floral reflectance spectra of petals from meadows and outcrops depicting blue chroma extremes.
**Figure S2:** Plots of pollen loads and proportion *Phacelia dubia* pollen for most common pollinator families.
**Figure S3:** The relationship between the proportional abundance of seven most common families, and population average blue chroma.

## Data Availability

All data and code used in this manuscript have been uploaded to Zenodo: https://zenodo.org/records/21842116.
