# Supplementary material for: The Most Effective Pollinators Predict Intraspecific Floral Color Diversity Across an Edaphic Matrix
Source: Ecol Evol. 2026 Sep 17;16(9):e74360. doi: 10.1002/ece3.74360 (PMC13585492; doi:10.1002/ece3.74360)
Supplement: Supplementary file 1 [file ECE3-16-e74360-s001.pdf]

Supplementary information for *The most effective pollinators predict intraspecific floral color diversity across an edaphic matrix*

Table S1: Location of each *Phacelia dubia* populations from which we collected data on floral phenotypes in the field and/or greenhouse, and data on pollinator community composition.

State	Habitat	Population ID	Latitude	Longitude	Field Phenotypes	Pollinators Collected	Greenhouse Phenotypes
AL	Meadow	VC	32.407692	-85.944282	Y	Y	Y
AL	Meadow	PR	32.453187	-85.795822	Y	Y	Y
AL	Meadow	KP	32.584823	-85.54402	Y	Y	Y
GA	Meadow	OM	32.84194	-83.606795	Y	Y	N
SC	Meadow	GM	33.046893	-81.26402	Y	Y	Y
SC	Meadow	CB	33.163497	-81.164811	Y	Y	Y
SC	Meadow	FF	34.595735	-81.424612	Y	Y	Y
SC	Meadow	CH	34.723273	-81.240635	Y	Y	Y
SC	Meadow	BC	34.735165	-83.274293	Y	Y	Y
AL	Outcrop	RR	32.63553	-85.576076	Y	Y	Y
AL	Outcrop	AO	32.65952	-85.525814	Y	Y	Y
AL	Outcrop	WM	32.971404	-85.342162	Y	Y	Y
GA	Outcrop	AP	33.513721	-82.269953	N	N	Y
GA	Outcrop	SS	33.525064	-82.251424	Y	Y	Y
GA	Outcrop	HR	33.542387	-82.256512	Y	N	N
GA	Outcrop	CM	33.7067	-83.927691	Y	Y	Y
SC	Outcrop	LR	34.460824	-80.748646	Y	Y	Y
SC	Outcrop	WTD	34.857905	-81.555221	Y	Y	Y
SC	Outcrop	OC	35.12657	-81.202162	Y	Y	Y

Table S2: Pollinators used to measure pollination efficiency metrics.

Family	Taxon	Number swabbed
Andrenidae		
	Andrena barbara	3
	Andrena miserablis	4
	Andrena sp.	5
	Andrena nasonii	1
	Andrena crataegi	2
Syrphidae		
	Eupeodes americanus	5
	Toxomerus geminatus	6
	Toxomerus marginatus	1
Apidae		
	Apis mellifera	5
	Ceratina sp.	1
Megachilidae		
	Osmia taurus	1
	Osmia lignaria	3
	Osmia georgica	2
	Hoplitis sp.	1
	Megachilidae sp.	1
Halictidae		
	Lasioglossum coeruleum	1
	Lasioglossum gemmatum	3
	Lasioglossum imitatum	2
	Lasioglossum sp.	1
Faniidae		
	Fannia sp.	4
Buprestidae		
	Acmaeodera tubulus	5

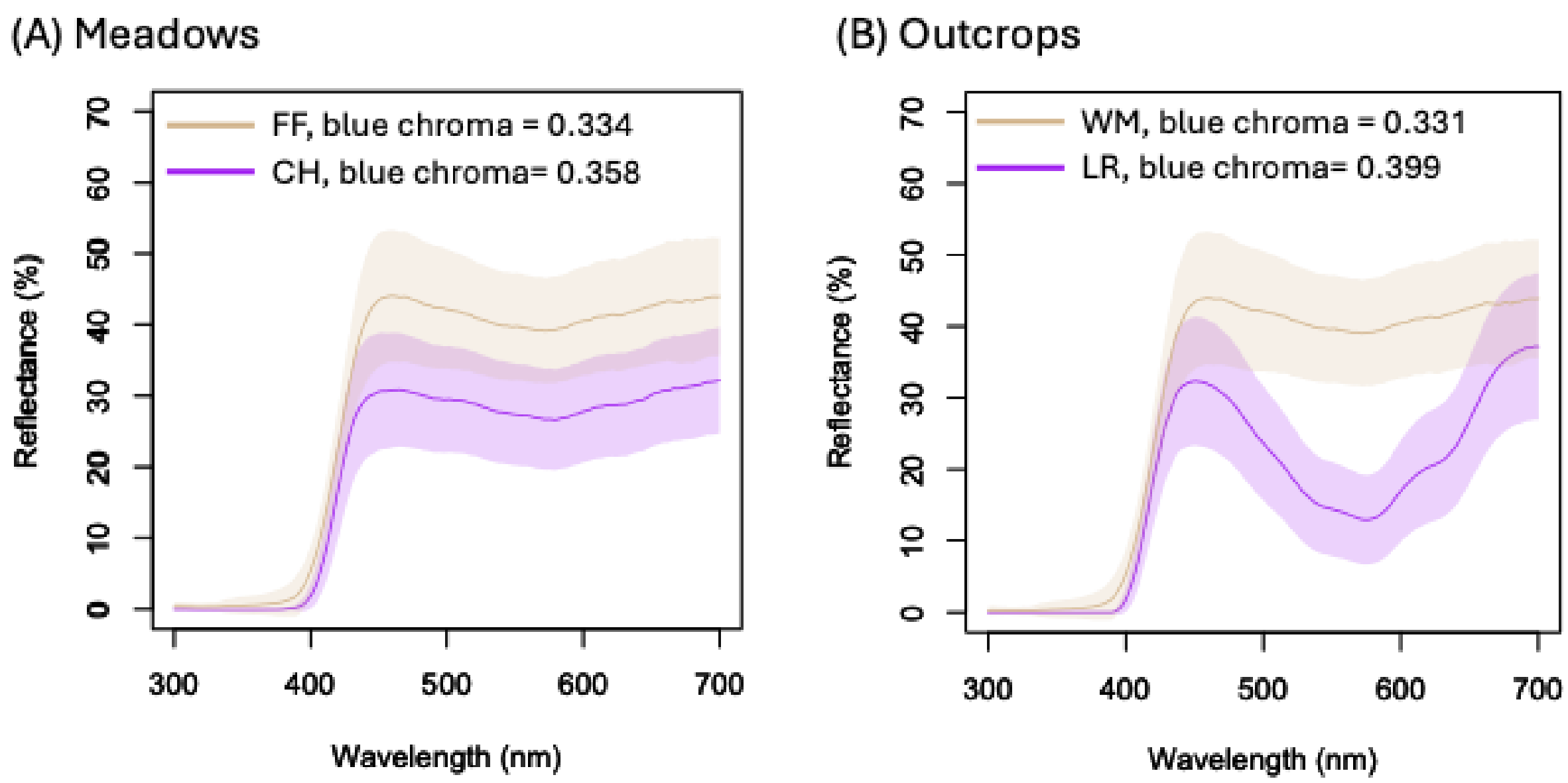

Figure S1: Reflectance spectra of petals from (A) meadow, and (B) outcrop populations with the lowest and highest average blue chroma for each habitat. Lines represent average reflectance and shaded areas represent the standard deviation.

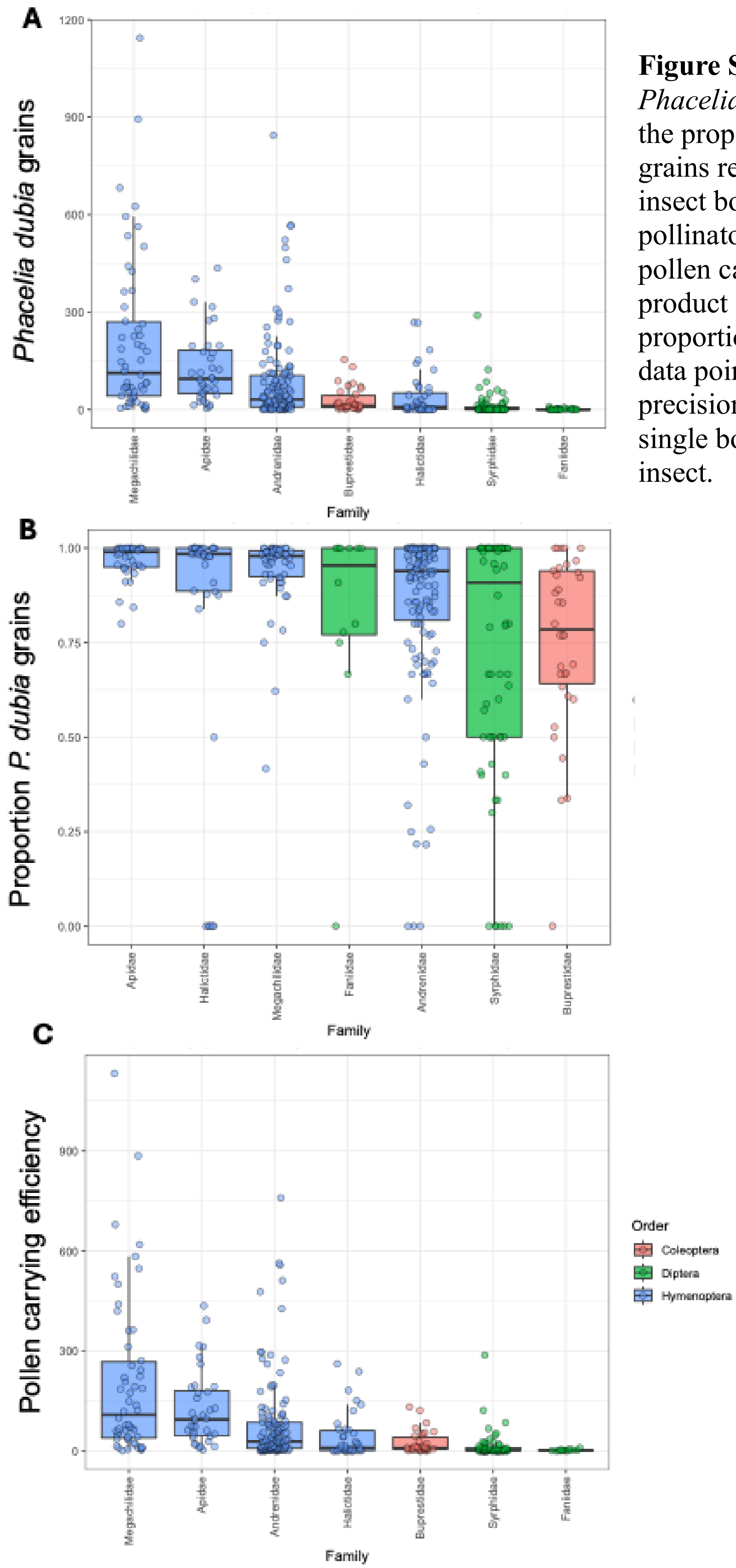

Figure S2: (A) the number of *Phacelia dubia* pollen grains and (B) the proportion of *P. dubia* pollen grains relative to all pollen grains on insect bodies for the 7 most abundant pollinator families of *P. dubia*. (C) pollen carrying efficiency was the product of *P. dubia* grains and the proportion of *P. dubia* grains. Each data point represents data from a precision glycerin jelly swab of a single body part on a field-collected insect.

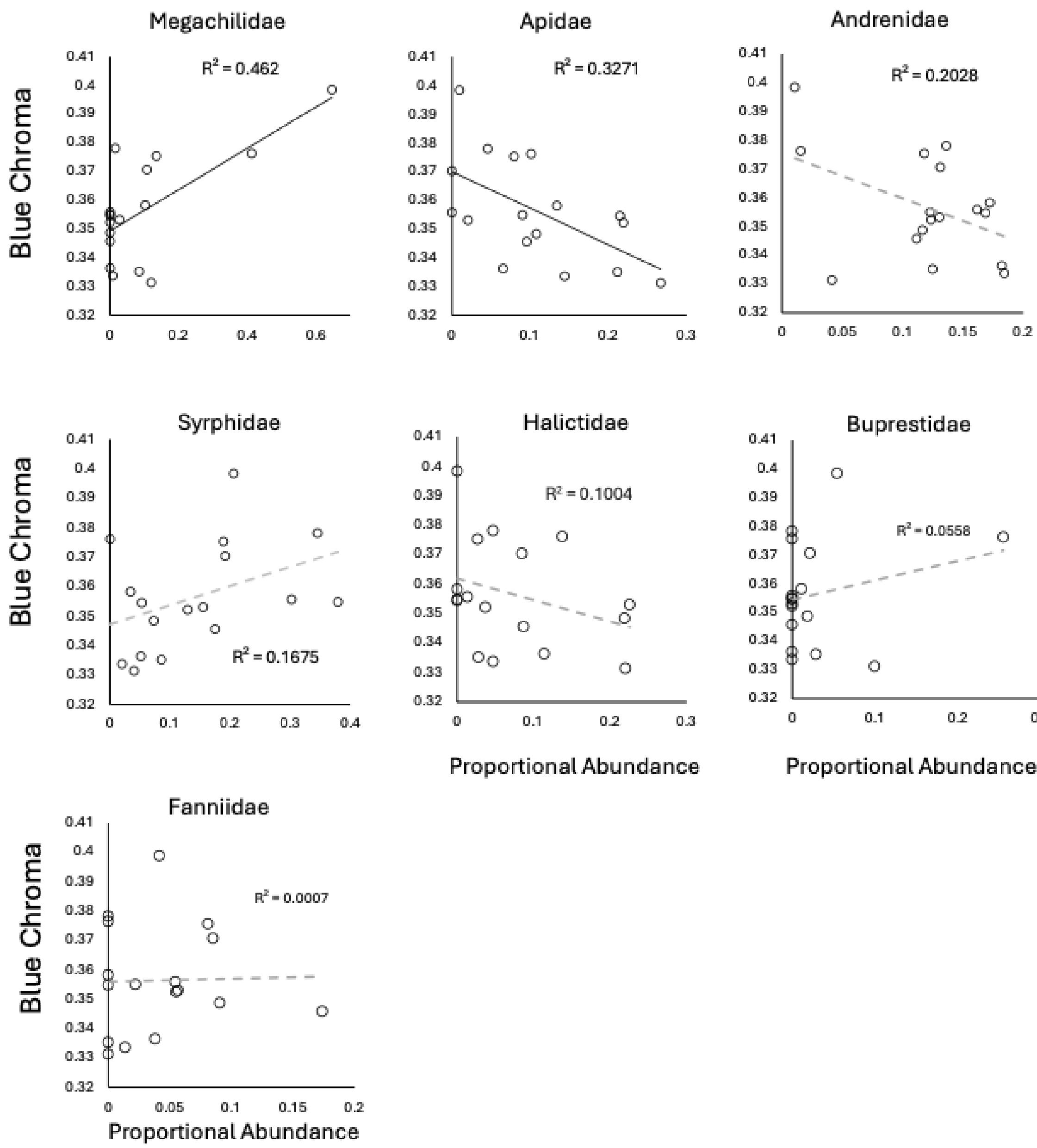

Figure S3: The relationship between the proportional abundance of each of the seven most abundant insect families, and population average blue chroma. Solid lines indicate significant linear relationships at $P<0.05$, and dashed lines represent nonsignificant trends. Plots are ordered by the proportion of variance in blue chroma explained. Note that X-axes differ in their range across each panel.
